# A Phylogeny-Based Benchmarking Test for Orthology Inference Reveals the Limitations of Function-Based Validation

**DOI:** 10.1371/journal.pone.0111122

**Published:** 2014-11-04

**Authors:** Kalliopi Trachana, Kristoffer Forslund, Tomas Larsson, Sean Powell, Tobias Doerks, Christian von Mering, Peer Bork

**Affiliations:** 1 Structural and Computational Biology Unit, European Molecular Biology Laboratory, Heidelberg, Germany; 2 Developmental Biology Unit, European Molecular Biology Laboratory, Heidelberg, Germany; 3 Max-Delbruck-Centre for Molecular Medicine, Berlin, Germany; 4 Institute for Systems Biology, Seattle, WA, United States of America; 5 Institute of Molecular Life Sciences, University of Zurich and Swiss Institute of Bioinformatics, Zurich, Switzerland; Swiss Federal Institute of Technology (ETH Zurich), Switzerland

## Abstract

Accurate orthology prediction is crucial for many applications in the post-genomic era. The lack of broadly accepted benchmark tests precludes a comprehensive analysis of orthology inference. So far, functional annotation between orthologs serves as a performance proxy. However, this violates the fundamental principle of orthology as an evolutionary definition, while it is often not applicable due to limited experimental evidence for most species. Therefore, we *constructed high quality "gold standard" orthologous groups* that can serve as a benchmark set for orthology inference in bacterial species. Herein, we used this dataset to demonstrate 1) why a manually curated, phylogeny-based dataset is more appropriate for benchmarking orthology than other popular practices and 2) how it guides database design and parameterization through careful error quantification. More specifically, we illustrate how function-based tests often fail to identify false assignments, misjudging the true performance of orthology inference methods. We also examined how our dataset can instruct the selection of a “core” species repertoire to improve detection accuracy. We conclude that including more genomes at the proper evolutionary distances can influence the overall quality of orthology detection. The curated gene families, called Reference Orthologous Groups, are publicly available at http://eggnog.embl.de/orthobench2.

## Introduction

Currently, more than 6,868 complete bacterial genomes are available in public repositories (GOLD Database v4.0) [1]. This wealth of genomic data has increased our understanding of gene family evolution and how various genomic events (e.g. lineage-specific gene loss/duplication and gene fusion/fission) shape genome architecture and organization [2]–[5]. Such evolutionary and comparative studies rely on the analysis of orthologous genes (homologous genes that diverged at a speciation event) [6] and consequently on their robust identification. Orthology detection methods have become pivotal in transferring knowledge [7] from experimentally annotated proteins in model species to other non-studied species due to the tendency of orthologs to share equivalent molecular functions [8].

However, the identification of genome-wide sets of orthologs for a large number of distantly related organisms is an enormous task due to the complex evolutionary history of gene families shaped by multiple events, such as horizontal gene transfer (HGT), gene duplication, gene loss or gene fusion. Often, an additional level of complexity rises due to protein domain reshuffling hindering the classification of full-length genes [9]. To systematically represent homology relationships between genes found in multiple species, Tatusov *et al.* [10] launched the concept of orthologous groups (COGs). Based on the original definition, these groups contain genes that have evolved from a single ancestral sequence present in the last common ancestor (LCA) of the species being compared, through a series of subsequent species divergence and gene duplication events. In addition to the mostly manually curated, and thus irregularly updated, COG database [11], there are other orthology resources that have adapted this definition of orthologous groups (i.e. eggNOG [12], OrthoDB, [13], OrthoMCL [14]). Yet, they may use different sequence similarity search tools, graph-based mining methods (i.e. triangulation vs. Markov clustering) or extra database features (i.e. eggNOG provides hierarchical orthologous groups). On the other hand, there are orthology projects that except of the distinct algorithmic differences define also alternatively what is an orthologous group (i.e. OMA [15], MultiParanoid [16]) or specify orthology after comparing gene family trees with an evolutionary model, such as a species tree (TreeFam [17], EnsEMBL Compara [18], PhylomeDB [19], LOFT [20], Homologene [21]). Kristensen *et al* [22] recently reviewed in detail the recent developments in the field and differences between the graph- and tree-based methodologies. This plethora of resources often overwhelms the users, who want to know which the most appropriate database for their research. However, any direct comparison between different inference algorithms is daunting due to 1) dissent among researchers concerning the definition of orthologous groups in multiple species comparisons and 2) the lack of a consensus set of species (*common proteomes*). The latter is particularly important as orthology is always defined relative to the most recent common ancestor (speciation event) of the genomes under consideration, i.e. the same gene can be characterized as an ortholog or paralog to other genes depending on whether more strictly or more broadly defined taxonomic groups are considered [5]. By promoting community standards, the “Quest for Orthologs” (QfO) consortium have proposed a reference species repertoire and at the same time, tries to overcome certain practical challenges. For instance, they have been successful to deal with the lack of a consensus on file formats supporting a unified orthoxml format [23]. This will definitely facilitate the comparative evaluation of tools and databases. In any case, a direct database comparison can only demonstrate the relative performance of algorithms/methodologies, rather than quantify the false discovery rate of each database. To achieve an absolute validation of orthology assignments, we need to reconstruct the evolutionary history of gene families (phylogenetic analysis) and manually curate a “gold standard” set of orthologous groups [23].

So far, the majority of benchmarks are either based on the functional conservation of detected orthologs - e.g. experimentally verified functional annotations, gene expression, enzymatic activity or other genomic features, such as gene order conservation [24]–[27]. However, these approaches neglect two important factors: 1) orthology is strictly defined in terms of the evolutionary history of a gene, and therefore is at best indirectly linked to the conservation of function [5], [8] and 2) the majority of sequenced species lack experimentally validated functional annotation, making them ineligible for such a benchmark [29]. Alternatively, there are statistical approaches, such as metaPhors [28] or latent class analysis [25], which use multiple projects to achieve statistical power and model the error for orthology inference. Still, they rely and recycle data from very different projects as we describe above and do not address the validation problem directly. Taking all above into account, a manually curated phylogeny-based benchmark test (“gold standard”) is more appropriate and can, in principle, be applied to any species. Initially, Altenhoff and Dessimoz [26] used manually curated trees from literature to validate 12 different orthology projects. While, recently, two independent studies tried to elaborate the concept of phylogeny-based benchmarking by manually curating the evolutionary histories of 70 metazoan- and 3 eukaryotic-specific families, respectively, and demonstrated a comparison between select databases and the benchmark set [30], [31].

Here, we present the first phylogeny-based benchmark for 238 bacterial species. We constructed a high quality “reference” orthologous groups (RefOGs) through intensive manual curation: inspecting multiple sequence alignments, evaluating the quality of phylogenetic trees and removing false assignment of orthologs. The main objective of our study is not the systematic analysis of orthology databases, as there is a poor overlap between the species repertoires of existing databases, as well as the dataset presented. Instead, we aim to demonstrate the benefits of phylogeny-based benchmark sets: 1) to disconnect orthology validation from gene function, which can have a great impact for functional genomics and 2) to explore the impact of various technical parameters (i.e. species selection) on the robustness of inference.

## Results

### A “gold standard” set of orthologs for bacterial species by phylogenetic analysis and manual curation

**Figure 1:**
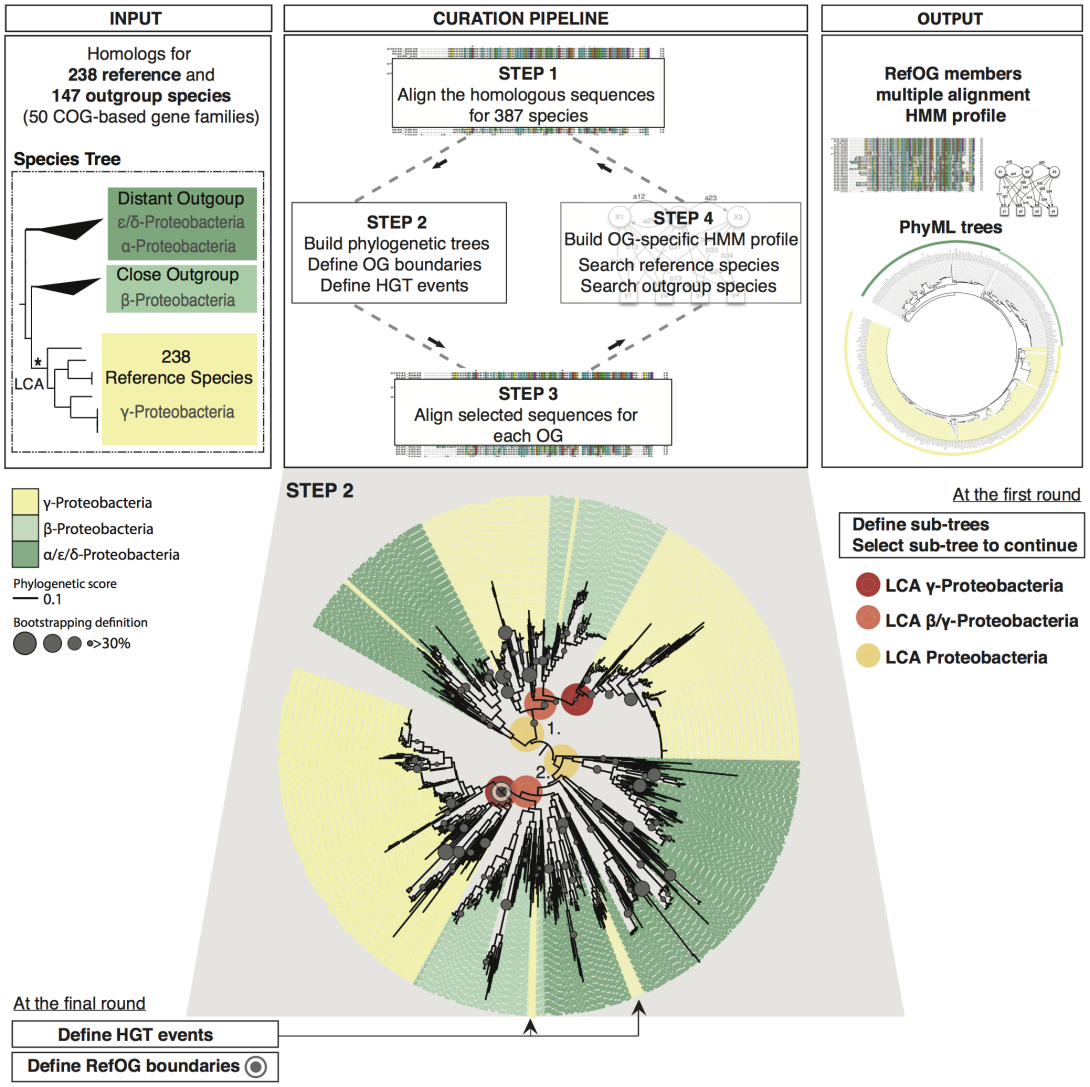
Flowchart for generating reference orthologous groups. The initial material (INPUT) is the homologs in 385 bacterial species for 50 error-prone families (Table S1 in Data S1). The homologs were chosen from the Clusters of Orthologous Groups (COGs), as these are inferred in the eggNOG database [10]. Depending on the complexity of the gene family, we performed two (ancient, well-conserved families) up to five (genus-specific, highly versatile families) rounds of curation. We followed four distinct steps at every round. The homologous (or later orthologous) sequences were aligned (Step 1) and the multiple sequence alignments (MSA) were used to build phylogenetic trees (Step 2). Each family tree was compared to a well-accepted species tree [32]. At the first round, species topology was used to define the boundaries of gene sub-families (sub-tress that include sequences from all three clades: α-, β- and γ- Proteobacteria), while at the following rounds, bona-fide orthologous groups related to the LCA of γ- Proteobacteria. The protein sequences of the members of each orthologous groups were re-aligned (Step 3). The new alignments were used to build Hidden Markov Models (HMMs), search the 385 bacterial genomes and define new homologs (Step 4). At the final round, we finalized the reference orthologous groups (OUTPUT) and identified and annotated through manual inspection HGT events and outgroups.

As mentioned above, identification of genome-wide sets of orthologous groups for a large number of distantly related organisms is an arduous task due to the complexity of the genomic events that shape the evolution of gene families. We chose to delineate the evolutionary history of 49 bacterial gene families (19 universal families that are also found in eukaryotes [32] and 30 bacterial-specific families) that can exemplify complex phylogenetic scenarios resulting from HGT, lineage-specific gene loss and other such events (Table S1 in Data S1). All 49 families correspond to a COG (Clusters of Orthologous Groups) that is a high-quality material given that the COG assignments are further checked and curated by hand to eliminate potential false-positives. The eggNOG algorithm uses COGs as starting material and builds up-to-date versions that include newly sequenced genomes. We used the eggNOG version of COGs as “homology seeds” for this study. We had to focus in a defined taxonomic clade to avoid technical challenges due to the large number of bacterial sequences in every COG (i.e. computationally expensive phylogenetic trees, or increased sensitive to noise and biases i.e. sequences may evolve at very fast rates in some clades of the Tree of Life or even within bacteria phyla leading to misalignments [33]–[35]). We decided to generate phylogenetic trees that describe the orthology relationships of the 49 aforementioned families in the gamma-Proteobacteria clade (238 genomes) for several reasons: 1) they represent one third of all bacterial genomes sequenced [1], [32], 2) there is an extensive research about their contribution in ecosystems and human health, and 3) there are well-established species relationships within the Proteobacteria clade, which facilitate the use of alpha- and beta-species as outgroups [32], [48], [49]. This is crucial for our analysis. As we mention above, orthology is defined relative to the common ancestor of the compared species. The phylogenetic information in the Proteobacteria clade allows us to define the boundaries of the orthologous groups and to place the last common ancestor of the gamma-proteobacteria in the family trees (Figure 1). We performed a three-step curating protocol (Figure 1) for all families, certifying that the curated orthologous groups (termed reference orthologous groups –RefOGs): 1) are not biased towards our initial collection of proteins (COG members based on eggNOG version 3) and 2) depict the evolutionary history of these gene families as accurately as possible. The final dataset consists of 4,698 reference orthologs in 49 RefOGs for hundreds of bacterial species.

### Function-based tests mask false assignments that can be revealed through phylogenetic-analysis

**Figure 2:**
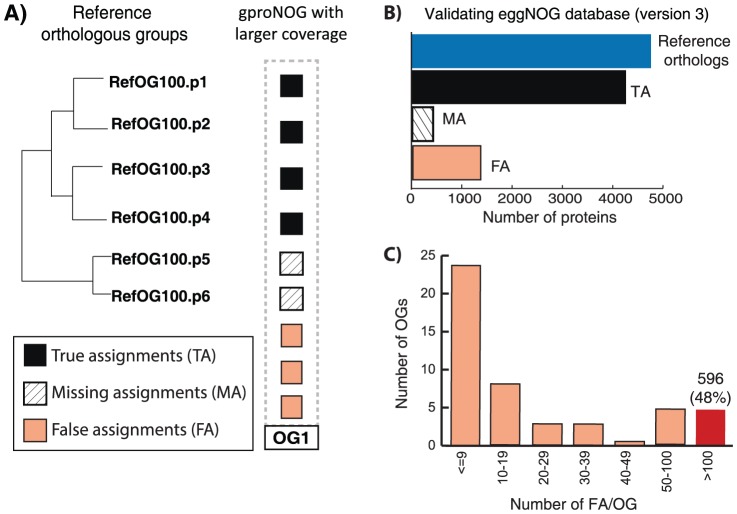
Benchmarking eggNOG database. A) To evaluate the performance of the database, we map the members (p1-p6) of every reference orthologous group (i.e. RefOG100) to the predicted orthologous groups and use the orthologous group with the highest coverage (i.e. OG1). Three classes of assignments are defined using OG1 orthology predictions: True assignments (TA) are the orthologs that have been grouped correctly in the database (black box). Missing assignments (MA) are the reference orthologs that were incorrectly excluded by the method (white stripped box). False assignments (FA) are those predictions that have been grouped in OG1, but are not reference orthologs (light red box). B) The number of true, false and missing assignments for eggNOG gamma-proteobacteria-specific orthologous groups (gproNOGs) applying the aforementioned scoring scheme. C) Distribution of FA per orthologous group. Half of the orthologous groups have less than 10 false assigned proteins (< = 9), contributing in less than 10% of this error category. The red box highlights five families that contribute to the ∼50% of the FA.

**Figure 3:**
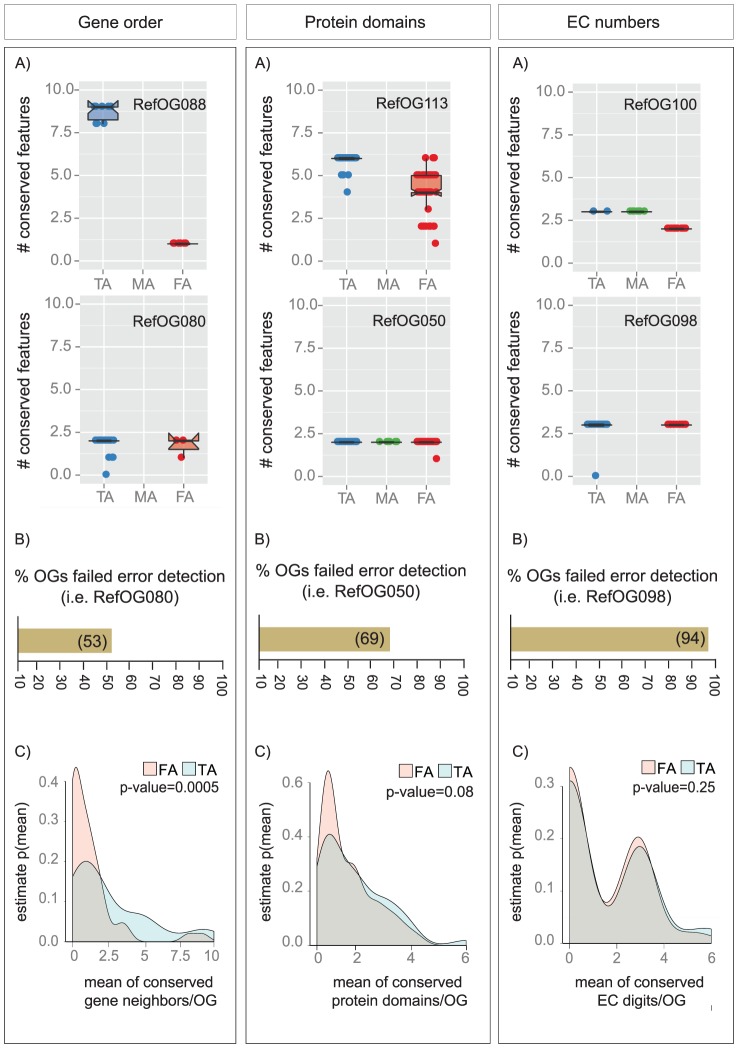
Function-based benchmark tests do not separate between false- and true- assignments. Consensus functional annotations were determined based on (i) gene order *(number of neighbor gene families that are conserved across RefOG members)*, (ii) protein domain content *(number of protein domains that are conserved across RefOG members)* and (iii) enzymatic activity *(number of EC digits that are conserved across RefOG members)* for every RefOG (Material and Methods). A) The distribution of conserved features across true-, missing- and false assignments for each family are illustrated with boxplots. The upper and lower boxplot panels exemplify families where the functional feature does or does not discriminate, respectively, false and true assignment. B) Bar plots show the number of orthologous groups that would be classified as *“accurately inferred”* using function-based tests. C) Density plots illustrate the probability to discriminate the true-, missing- and false-assignments for every function-based test (density of mean number of the conserved features for every assignment category/RefOG). The data for all 49 RefOGs is shown in the Figures S1-S3 in File S1 and Tables S2-S4 in Data S1.

To demonstrate how our benchmark dataset can be used to validate orthology inference and to compare its performance with function-based tests, we use eggNOG, the in-house orthology database [12]. To allow meaningful evaluation of an orthology database, the orthologs (or orthologous groups) should be inferred at the same phylogenetic level as the RefOGs. Accordingly, we mapped the members of each RefOG to the eggNOG orthologous groups for gamma-proteobacteria (gproNOGs) and classified the predicted orthologs into three different categories: 1) true assignments (orthologs have been grouped correctly), 2) false assignments (proteins included in the eggNOG orthologous group, which are not reference orthologs) and 3) missing assignments (reference orthologs which were left out in eggNOG) (Figure 2A). In total, we identified 4359, 1374, and 429 proteins in each of these categories, respectively. eggNOG correctly clusters 91% of the reference orthologs, but also accumulates a considerable number of false assignments. A closer look at our comparison reveals that almost half of the total false assignments (∼600 out of the 1374 proteins) are accumulated mainly in 5 of the 49 orthologous groups (Figure 2B, Table S2 in Data S1) In all cases, the true and false assignments share common protein domains, e.g. gproNOG00600 (corresponds to RefOG075) shows how the Glt symporter domain (Pfam: PF0316 [36]) supports the grouping of 158 proteins that we can clearly separate in our phylogenetic analysis. In other words, the protein domain content of orthologs that serves commonly as a validation test of prediction [25], [26] and as a function proxy [9], [37], would classify all five orthologous groups as correct. However, this phylogeny-based test exposes functionally related, false-positive assignments and therefore enables a more accurate database evaluation. To quantify the frequency of such cases, where function-based tests fail to correctly validate the automated orthology predictions compared to our novel phylogeny-based test, we investigated if false- and missing- assignments can be differentiated based on three functional/genomic features: 1) gene order, 2) protein domain content, and 3) Enzyme Commission (EC) numbers. We limited our comparison to these three attributes, as not all proposed tests [24]–[27] are applicable in the case of gamma-proteobacteria. We retrieved each of these features (Tables S3–S5 in Data S1) for every protein classified as true, false or missing orthologs (Material and Methods). Each function-based test works well for capturing missing assignments, but the phylogeny-based test outperforms function-based tests in identifying false assignments (Figure 3; Figures S1–S3 in File S1). It is clear that many false assignments will be considered “true” orthologs if evaluated only on the basis of these functional features, reflecting the limitations of function-based tests. A closer (manual) inspection of the false assignments (i.e. analyzing alignment quality and the phylogenetic trees built for the families) further justifies the phylogeny-based validation (data not shown). Among these three validation factors, gene order can identify more accurately the false assignments, illustrating again the need to combine orthology predictions with synteny information where available [27], [38]–[40].

### A phylogeny-based benchmark test can point out false assignments resulting from HGT events

Gene families usually expand through duplication and horizontal gene transfer (HGT), resulting in paralogs (duplication-derived homologs) and xenologs (HGT-derived homologs), and can contract through gene loss or pseudogenization [5]. HGT, a prominent mechanism of genome evolution mainly in unicellular species, can be identified only through phylogenetic analysis [41], [42]. To the best of our knowledge, no existing orthology identification approach explicitly removes xenologs from the predicted orthologous groups. Previous studies using simulated data [43], [44] have demonstrated that HGT disrupts all orthology inference methods, while analysis of the latest version of the arCOG database, based on 120 archeal genomes, revealed that single-gene gain patterns through HGT are prominent and not completely random [45]. This evidence highlights the importance of identifying the xenologs and flagging them as false assignments derived from HGT events.

Although our benchmark set is restricted to only 49 families, it has the potential to identify xenologs, flag them as false assignments and occasionally cluster them separately. After manual inspection of the phylogenetic trees for the selected families, we identified 110 proteins (Table S6 in Data S1), which are nested within the close and distant outgroups and represent HGT events. After benchmarking, we counted 79 out of these 110 xenologs (∼5% of the false assignments) affect, in total, 19 eggNOG orthologous groups. This may be a conservative estimate, as we ignored putative HGT events located in the boundary between beta- and gamma-proteobacteria where it is hard to distinguish phylogenetic signal from random noise. Yet, it also shows that HGT occurs as well-delineated events that are in principle traceable and there is considerably more vertical than horizontal signal in all families studied.

To test if this particular type of error can be detected by function-based tests, we compared the protein domain content and gene order of the 105 xenologs to the true orthologs (true assignments). Only in 3 out of these 19 cases do the xenologs have different domain content compared to the rest of the members (Table S6 in Data S1). In all other cases, the domain content is well conserved even for xenologs (7 orthologous groups). In other words, 16 eggNOG orthologous groups would have been falsely considered accurate if evaluated using only this functional feature. On the other hand, since xenologs tend to be re-located in the genome, gene order can serve as an excellent detection tool. Indeed, the xenologs in 9 out these 16 eggNOG groups showed low conservation in their gene neighbors.

### Impact of species coverage on orthology calling

**Figure 4:**
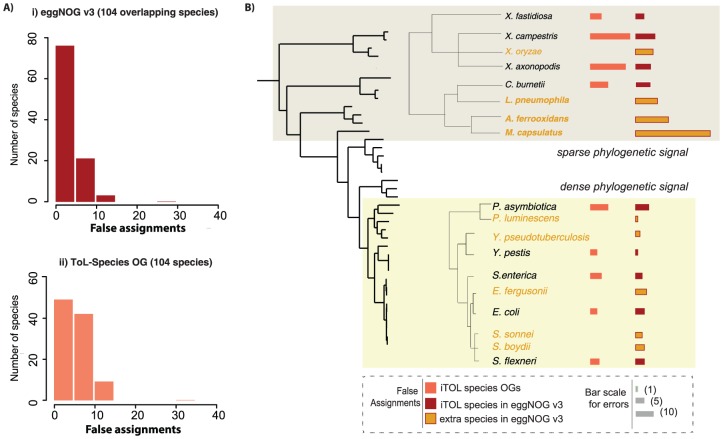
Species selection is the most influential factor for robust orthology inference. The in-house pipeline eggNOG was used to generate orthologous groups based on 104 gamma-Proteobacteria species that belong to the Tree of Life (ToL) –so called ToL-Species OGs. A) For each of the 104 species, we count the number of false assignments in the ToL-Species OGs and eggNOG database (v3). The distribution of errors between the two datasets is significantly different (p-value <<0.05, Kolmogorov–Smirnov test). B) Bar plots illustrate the species-specific contribution in the false assignments pool. Species with *“dense”* (taxonomical clades with a large number of closely related bacteria) *and “sparse”* (taxonomical clades with small representation) phylogenetic information show a different accumulation pattern (false assignments across all 49 RefOGs). Species in black letters exist both in ToL-Species OGs and public eggNOG (overlapping species), while species in orange letters indicate eggNOG-specific species (not present in the Tree of Life).

Our previous analyses using the animal-specific RefOGs revealed the importance of the species selection in orthology prediction and database performance [12], [30]. We observed that 1) expanding the number of animal species in consecutive eggNOG releases has improved our database and 2) species with long phylogenetic branches (i.e. *C.elegans*) tend to accumulate more errors. A careful inspection of the false assignments in this study revealed a similar pattern. Therefore, to investigate further this observation, especially since a substantial species collection (hundreds of bacterial species vs. tens of animal species) is available, we repeated a similar analysis using the bacterial-specific RefOGs and exemplifying how to select a species repertoire that most improves overall performance of an orthology inference method. Using the eggNOG pipeline, we generated two separate orthology datasets. The new datasets contain a smaller number of species; 104 and 152 species respectively, which overlap with the 238 proteomes of gamma-proteobacteria in the public eggNOG database. We refer to these two new datasets ToL-Species (Figure S2 in File S1) and ToL-Genus (Figure S3 in File S1), respectively, as they map at the species and genus level with the gamma-Proteobacteria that are present in the available Tree of Life (ToL) [32]. All three datasets have the same phylogenetic range, meaning that the LCA of the set of the included species is the same phylogenetic node, but they have different species coverage. When mapping each dataset against the reference orthologs for the overlapping 104 species, we observe a 1.4-fold increase in false assignments for the smaller datasets over eggNOG version 3 (Figure 4A, Table S7 in Data S1). Although we observed the opposite result in the category of missing orthologs (smaller datasets perform better) (Figure S4 in File S1), the cumulative error distribution (false and missing orthologs) differs significantly (p-value <<0.05, Kolmogorov–Smirnov test) and favors the more inclusive dataset (eggNOG v3) (Table S7 in Data S1). This suggests that the additional species enable the correct identification of orthology clusters by increasing the amount of available phylogenetic signal. After scrutinizing the data, we concluded that there is one frequent source of error: the in-paralogs calling. The latter is an important step in many graph-based methodologies, including eggNOG. For closely related species/genera (“short phylogenetic distance”), i.e. *Escherichia* and *Salmonella* (Figure 4B, Table S8 in Data S1), we tend to detect bona-fide in-paralogous groups (paralogs derived via duplication after the *Escherichia*-*Salmonella* speciation event). Often, missing orthologs represent such bona-fide groups that fail to cluster together during the graph-mining step to define a gamma-Proteobacteria (deeper ancestor) orthologous groups, instead they cluster as a species/genus-specific cluster (Figure S5 in File S1). Alternatively, missing paralogs are strain-specific duplications followed by increased evolution rates that affects the sequence similarity between paralogs. This is an excellent example how this phylogeny-based benchmark dataset helped us to recognize a limitation of the current eggNOG algorithm and try to design a safety net in the next release. On the other hand, remote species (e.g. *Methylococcus*) accumulate a large number of false assignments (Figure 4B, Table S8 in Data S1). This stems from the opposite problem: the sequence similarity between remote species is low and thus, it is difficult to separate the out-paralogs (duplicated homologs preceding the speciation event).

## Discussion

The rapid accumulation of genomic data presents a challenge for accurate large-scale, genome-wide orthology inference and annotation projects. As orthology is essential for comparative and functional genomics, it is crucial to define and introduce well-accepted standards for evaluating the results of orthology annotation. We have thus generated a phylogeny-based benchmark set for assessing orthology predictions in bacteria, which neither relies on any functional information of the genes involved, nor is subject to the shortcomings of using conserved function as a proxy for common descent from a speciation event. It is essential to disassociate the technical validation of orthology inference from function in order to be able to leverage our knowledge in the field of functional genomics, as circularity otherwise hampers any conclusions that are drawn. The “ortholog conjecture” (orthologs with a similar degree of sequence divergence, are generally more conserved in function than paralogs) is a nice example. Although, this has been primarily supported by theory, available empirical studies are controversial. Systematic error quantification based on a “gold-standard” will allow the improvement of orthology inference and the study of functional evolution of proteins.

We used the latest version of gamma-proteobacteria-specific orthologous groups from the eggNOG database to demonstrate the advantages of RefOGs against previously used tests to 1) identify errors concealed to functional benchmarks, 2) deal with xenologs (falsely assigned orthologs stemming from HGT events) and 3) determine how species selection effects orthology reconstruction. This bacterial-specific benchmark set can be combined with our previously published animal-specific benchmark set. Currently, there are 119 publically available RefOGs (70 in eukaryotic and 49 in prokaryotic species with 19 spanning both domains of life). Similar to our previous findings –based on the animal-specific dataset [12], [30]- the bacterial-specific RefOGs demonstrate the importance of the species repertoire in orthology prediction. In both cases, the evolutionary-distant species (i.e. *C. elegans* or *A. baumannii*) accumulate the larger number of errors. At the same time, introducing more species improves the overall performance of the database; i.e. benchmarking the two more recent releases of eggNOG database against the animal RefOGs [12] or herein (Figure 4). Analyzing hundreds of bacterial species gave us the proper resolution to quantify the errors based on the density of selected species on the tree (short vs. long branches). To facilitate access to the curated benchmark families, we have created a web interface through which details on all RefOGs can be retrieved. Alignments, protein sequences, phylogenetic trees and sequence profile Hidden Markov Models (HMMs) for each RefOG can be downloaded and used for future analyses. These data are available under the Creative Commons Attribution 3.0 License at: http://eggnog.embl.de/orthobench2.

Finally, we need to stress the limitations of manually curated, phylogeny-based benchmarking tests. Despite all our effort to provide accurate RefOGs, a reference tree still remains a tentative model of the evolutionary history of the studied species. This implies that 1) update and maintenance are essential when novel related sequences/genomes and improved tree-building approaches become available and 2) the dataset is limited to the orthology relationships of the reference species. Regarding the former conclusion, we are committed to supporting this effort; for instance, we recently updated the metazoan dataset and investigated errors that were pointed out from users. Still, we welcome any community effort for this project: sequences derived from incomplete or erroneous gene models or questionable robustness of tree topologies after adding or removing species are challenges for a long-lasting dataset that should be tackled on a collaborative manner. The Quest for Orthologs (QfO) consortium has shown that the orthology community cares for standardization and quality control [23]. We anticipate that fostering this dataset and internalizing our insights on species selection can instruct how to best construct reference proteome collections to facilitate a fair and accurate comparison of the performance of different orthology inference methods. Right now, QfO consortium has decided on 66 Reference Proteomes spanning all three major domains of life, which implies that a phylogeny-based benchmarking dataset should be curated at the level of last universal common ancestor. This brings us to the second remark: a phylogeny-based test is only appropriate for the examined species. In principle, since we provide a phylogenetic tree for every RefOG, the users can benchmark orthologous groups for any subset of the reference species and delineate the orthology/paralogy (pairwise) relationships referred to every speciation event that is supported by the tree (i.e. LCA of Enterobacteriales). On the other hand, if a database provides orthologous groups for a more ancient speciation event (LCA of Proteobacteria), then our dataset is not appropriate anymore (i.e. all gamma-Proteobacteria in Figure 1 (yellow lines) should be clustered together, not only the selected RefOG).

In conclusion, we wholeheartedly support phylogeny-based benchmarking sets for orthology. As we demonstrated herein, they can provide guidance on inferring robust orthology predictions and disassociating function from the technical validation of orthology. Nevertheless, they have limitations that can be overcome through the collective efforts of this community.

## Materials and Methods

### Building the Reference Orthologous Groups (RefOGs)

Starting with orthologous groups at the level of last universal ancestor (COGs) in the eggNOG database (Table S1 in Data S1), we manually recovered orthologous groups for 238 gamma-proteobacterial species, which we refer to as Reference Orthologous Groups (RefOGs). To define the boundaries of each RefOG based on the last common ancestor (LCA) of gamma-proteobacteria, we used 147 genomes as outgroups (including species from beta- and alpha-proteobacteria as well as more distant species). Initially, the homologous sequences were aligned using MUSCLE [46] and the alignments were used to build aLTR-supported phylogenetic trees using PhyML version 3.0 [47]. For trees (i.e. groups) that represent multiple gamma-proteobacteria-specific families, we curated the family that was best resolved by the presence of the outgroup(s) taking into account well-accepted species trees [32], [48], [49]. However, in several cases no clear outgroup could be defined, hampering the resolution at the desired level. For these families, RefOGs were defined based on i) manual inspection of the alignments and ii) previously published descriptions of the families.

After the initial curation of the families, all sequences determined to be members of a particular gamma-proteobacteria orthologous group, as well as the corresponding outgroup sequences, were aligned using MUSCLE [46]. Alignments were cut based on the first and last well-aligned columns as estimated using GBLOCKS [50] with the following parameters: (Minimum Length Of A Block: 10, Allowed Gap Positions: With Half, Use Similarity Matrices: Yes). This was done in order to remove highly divergent N- and C-terminal parts of each alignment where misalignment is assumed to be common. Alignments were further manually cleaned to remove large parts where all sequences but one had gaps or short sequences that did not align within a conserved “block”. Based on the refined alignments, Hidden Markov Models (HMMs) were built using the HMMER3 package [51]. At a second refinement step, the HMM models were used to identify related sequences that were previously unidentified from the 385 aforementioned genomes. We did not define a global cut-off for sequence recruitment, but instead treated each family uniquely by adding all sequences with a bit score within the range of bit scores of already known members. After the addition of those sequences, phylogenetic trees were calculated using PhyML version 3.0 [47] with the following settings: 100 bootstrap replicates, optimization of tree topology, branch lengths and rate parameters, four substitution rate categories and the NNI topology search option. RefOG identifiers, alignments, HMM models and trees are available at http://eggnog.embl.de/orthobench2.

### Identifying the functional annotations for the reference orthologs and eggNOG orthologous group members

eggNOG [12] generates OGs for different taxonomic levels; thus, in the current comparison we used OGs generated for gamma-proteobacteria species only (i.e. gproNOGs). The predictions are publicly available at: http://eggnog.embl.de/version_3.0/downloads.html. We mapped the reference orthologs to the gproNOG orthologous groups (Figure 2). For the following analysis, each RefOG was associated with the gproNOG for which it would maximize the number of true orthology assignments. For every protein in the dataset, three distinct functional features were retrieved, using the following approach:

1. Domain content conservation analysis: Protein domain were assigned to the proteins by scanning them against the Pfam database (Pfam 26.0) [36] using HMMER3 [51] with curated family-specific score thresholds taken from Pfam. We then defined “family-specific conserved domains” for each RefOG by identifying protein domains that are present in more than 75% of its members. This cutoff was chosen conservatively to reflect the variability of domain content as found within gene families where most of the sequence is orthologous but where potentially a few domains can have different histories as a result of domain shuffling. Content conservation of a protein relative to its RefOG was then defined as the number of these “family-specific conserved domains” that it was found to contain. Since the analysis considers domain content rather than domain architecture (the set of unique domains rather than the vector of ordered domains), no special handling of short repeat-type domains was considered necessary.
2. Gene order conservation analysis: For each gene in the RefOGs, we inspected 10 adjacent genes (five before, five after, approximately matching the size of many bacterial operons). Although this number is significantly larger from the average operon size (3 genes) [52], it is more appropriate for larger phylogenetic distances that important genomic rearrangements may have taken place. This is also in agreement with previous studies that have identified important regulatory and functional genomic structures, named as gene clusters [53] or uber-operons [54], which are composed from larger genomic regions. Gene families were defined among the resulting total set of genes by linking together (through single linkage) all genes that are identified as homologs by NCBI BLAST [55] at an E-value threshold of 10e-5 and additionally required the aligned region to be at least 90% of the length of the shorter gene. The resulting clusters define (operationally) gene families in the immediate neighborhood of each RefOG member. These gene families were considered conserved (“family-specific conserved neighbors”) for a RefOG if they were present in the genomic neighborhoods of at least 75% of the genes in each RefOG. Gene order conservation for a particular protein was then defined as the number of these conserved families also found in its 10-gene genomic neighborhood.
3. Enzymatic activity conservation analysis: For each protein, its closest BLAST hit in version 66.0 of the KEGG database [56] was determined, in 99% of all cases this was to an identical sequence. Based on the KEGG pathway membership of these matches, and parsing the descriptions of the corresponding KEGG pathways for Enzyme Commission (EC) numbers, proteins were assigned EC number functional annotations. Given the hierarchical nature of EC numbers, annotations were expanded to include both more and less specific descriptions (e.g. EC:1.2.3.4 expanded to EC:1.2.3.4, EC:1.2.3.-, EC:1.2.-.-). We considered each of these descriptions to be conserved for a RefOG (“family-specific conserved enzymatic activity”) if assigned to more than 75% of its members. The functional conservation score for each protein was then defined as the number of these conserved functional terms assigned.

### Evaluating the effect of species selection on orthology inference

To investigate the impact of species selection, we used the eggNOG pipeline to generate two new datasets (available upon request). The new datasets, referred to as ToL-Species and ToL-Genus, are gamma-proteobacterial orthologous groups generated using only a subset of species included in version 3 of the eggNOG database. The Ref.Species dataset provides predictions for 104 gamma-proteobacterial proteomes found in the Tree of Life (ToL) resource [32] – a restricted species catalog compared to the publicly available eggNOG version 3. The ToL-Genus dataset provides orthologs for 152 gamma-proteobacteria that map at the genus level to the ToL species (e.g. *Xylella fastidiosa* is a ToL species and belongs both at the ToL-Species and ToL-Genus dataset, but *Xylella oryzae* exists only in the ToL-Genus dataset). Both the ToL-Species and ToL-Genus proteome are subsets of the set of 238 eggNOG gamma-proteobacterial species.

## Supporting Information

File S1 **Supporting figures.** **Figure S1**, Gene order as discriminator of false-, missing- and true-assignments. **Figure S2**, Enzymatic activity as discriminator of false-, missing- and true-assignments. **Figure S3**, Protein domain architecture as discriminator of false-, missing- and true-assignments. **Figure S4**, Error distribution for eggNOG v3 and ToL species-specific orthologous groups. **Figure S5**, Species-specific distribution of errors for the three different datasets of orthologous groups (ToL Species, ToL Genus & eggNOG).
(DOC)

Data S1 **Supporting tables.** **Table S1**, Information about the eukaryotic- and prokaryotic- Reference Orthologous Groups. **Table S2**, Benchmarking eggNOG v3 using OrthoBench. **Table S3**, Benchmarking eggNOG v3 using gene order. **Table S4**, Benchmarking eggNOG v3 using enzymatic activity. **Table S5**, Benchmarking eggNOG v3 using protein domain architecture. **Table S6**, Table of the Pfam domain composition for xenologs and orthologs (true assignments). **Table S7**, Benchmarking eggNOG v3, ToL Species, and ToL Genus orthologous groups using the reference orthologs for the 104 overlapping species. **Table S8**, Table of the error distribution of each species at the gene- and group-level.
(XLS)
