# Supplementary material for: A Phylogeny-Based Benchmarking Test for Orthology Inference Reveals the Limitations of Function-Based Validation
Source: PLoS One. 2014 Nov 4;9(11):e111122. doi: 10.1371/journal.pone.0111122 (PMC4219706; doi:10.1371/journal.pone.0111122)
Supplement: File S1 — Supporting figures. Figure S1, Gene order as discriminator of false-, missing- and true-assignments. Figure S2, Enzymatic activity as discriminator of false-, missing- and true-assignments. Figure S3, Protein domain architecture as discriminator of false-, missing- and true-assignments. Figure S4, Error distribution for eggNOG v3 and ToL species-specific orthologous groups. Figure S5, Species-specific distribution of errors for the three different datasets of orthologous groups (ToL Species, ToL Genus & eggNOG). (DOC) [file pone.0111122.s001.doc]

Supplementary File S1

**A phylogeny-based benchmarking test for orthology inference reveals the limitations of function-based validation**

Kalliopi Trachana*^4^, Kristoffer Forslund*^1^, Tomas Larsson^1,2^, Sean Powell^1^, Tobias Doerks^1^, Christian von Mering^5^, Peer Bork ‡^1,3*^

‡ Corresponding author contact information: [bork@embl.de](mailto:bork@embl.de)

* These authors contributed equally to this work

Figure S1: Gene order as discriminator of false-, missing- and true-assignments. Using functional for every reference orthologs, we defined a consensus annotation for each RefOG. Boxplots show how gene order information is distributed in FA, TA and MA. Using this feature has only 50% chances to discriminate between FA and TA (Table S3).


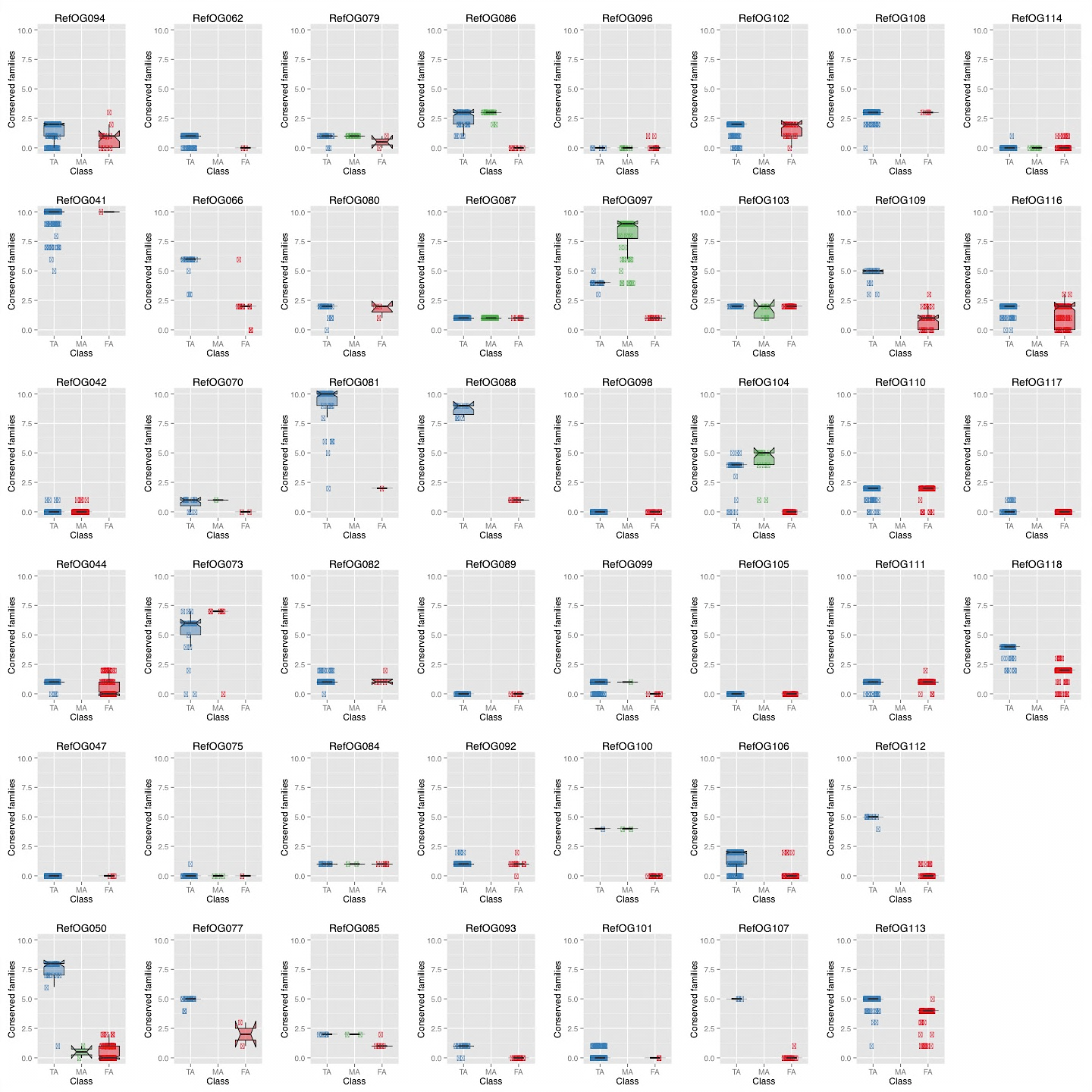


Figure S2: Enzymatic activity as discriminator of false-, missing- and true-assignments. Using functional for every reference orthologs, we defined a consensus annotation for each RefOG. Boxplots show how EC information is distributed in FA, TA and MA. Using this feature has only 6% chances to discriminate between FA and TA (Table S4).


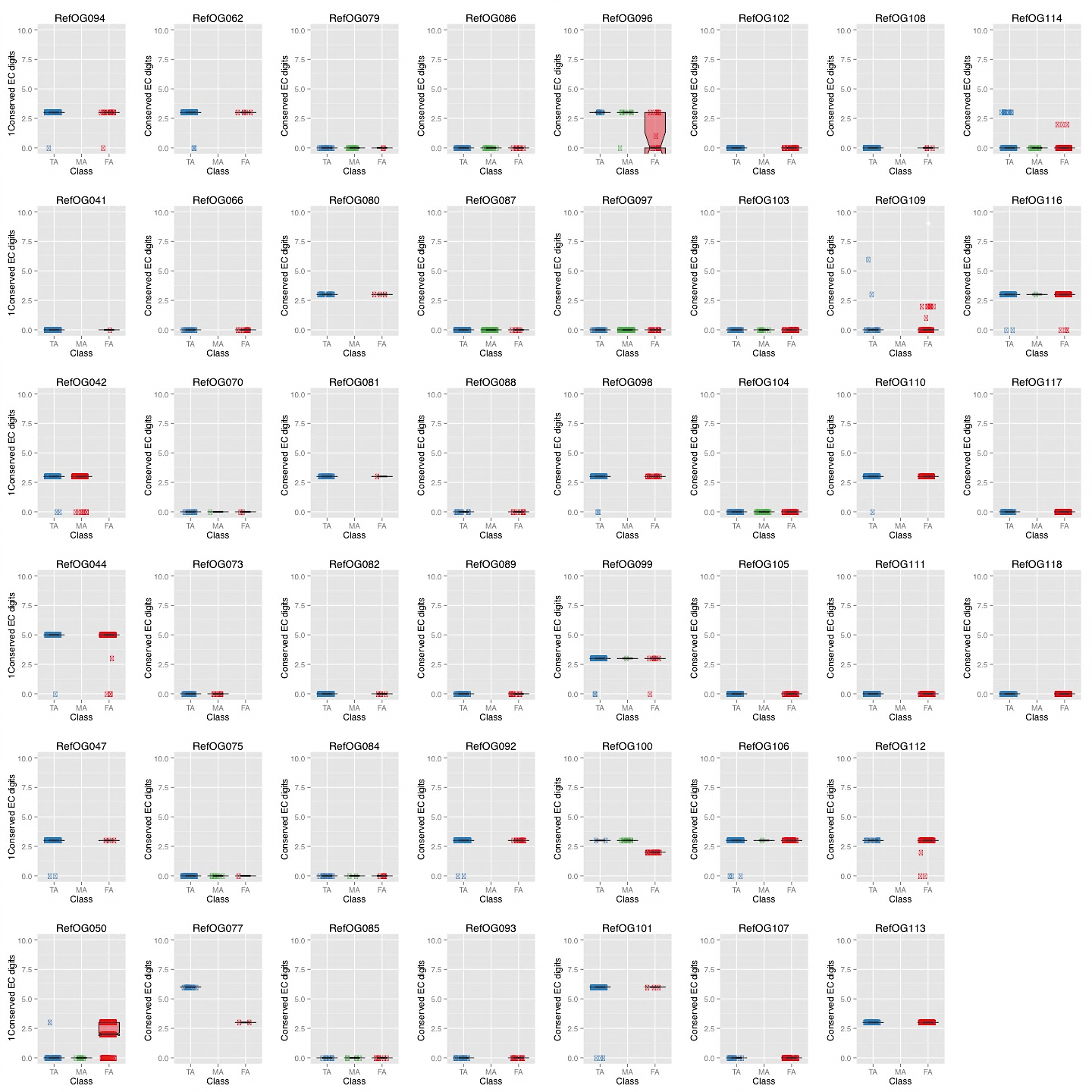


Figure 3: Protein domain architecture as discriminator of false-, missing- and true-assignments. Using functional for every reference orthologs, we defined a consensus annotation for each RefOG. Boxplots show how protein domain information is distributed in FA, TA and MA. Using this feature has only 30% chances to discriminate between FA and TA (Table S5).


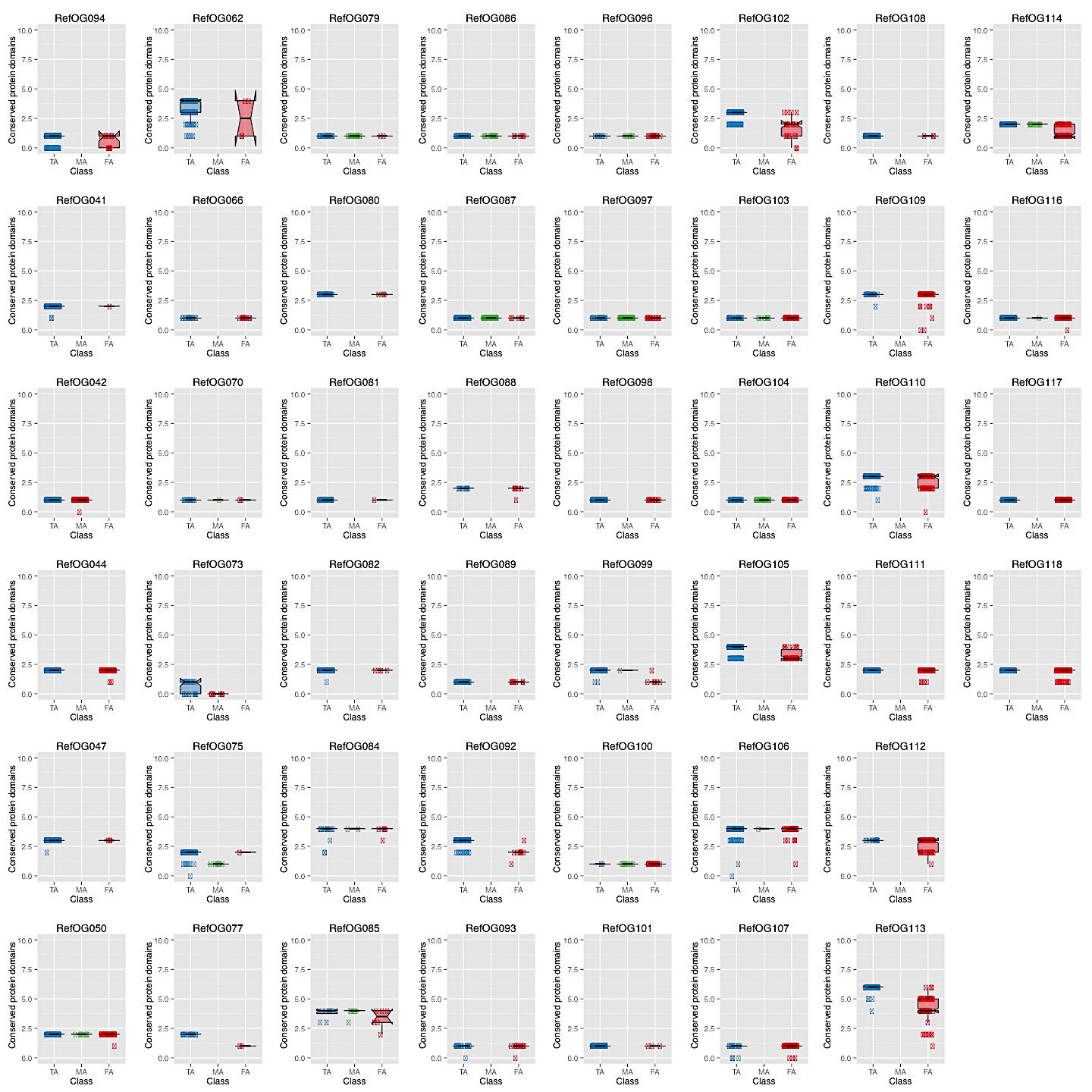


Figure S4: Error distribution for eggNOG v3 and ToL species-specific orthologous groups.

**Figure S5: Species-specific distribution of errors for the three different datasets of orthologous groups (ToL Species, ToL Genus & eggNOG).** This figure illustrate the same tree structure as Figure 4 including the ToL Genus dataset. We use the species tree to define species clusters with dense (i) –species are separated by short branches- and sparse (ii) –species are separated by long branches- phylogenetic distribution. To evaluate the impact of species density and species distribution, we compared 3 different datasets: 1) ToL Species, 2) ToL Genus and 3) eggNOG. eggNOG (dataset with the higher species density) outperforms the two other datasets. i) Dense area: Increasing the number of species (within the same genus) reduces the number of FA. ii) Sparse area: Increasing the number of genus (i.e. including *Oceanospirillales* – light blue line) clearly optimize the orthology prediction. Black, regular letters indicate species that are present in all datasets.
